# Application of Light Scattering Techniques to Nanoparticle Characterization and Development

**DOI:** 10.3389/fchem.2018.00237

**Published:** 2018-06-25

**Authors:** Patrícia M. Carvalho, Mário R. Felício, Nuno C. Santos, Sónia Gonçalves, Marco M. Domingues

**Affiliations:** Instituto de Medicina Molecular, Faculdade de Medicina, Universidade de Lisboa, Lisbon, Portugal

**Keywords:** nanoparticles, dynamic light scattering, zeta-potential, antimicrobial peptides, anticancer peptides, cardiovascular diseases

## Abstract

Over the years, the scientific importance of nanoparticles for biomedical applications has increased. The high stability and biocompatibility, together with the low toxicity of the nanoparticles developed lead to their use as targeted drug delivery systems, bioimaging systems, and biosensors. The wide range of nanoparticles size, from 10 nm to 1 μm, as well as their optical properties, allow them to be studied using microscopy and spectroscopy techniques. In order to be effectively used, the physicochemical properties of nanoparticle formulations need to be taken into account, namely, particle size, surface charge distribution, surface derivatization and/or loading capacity, and related interactions. These properties need to be optimized considering the final nanoparticle intended biodistribution and target. In this review, we cover light scattering based techniques, namely dynamic light scattering and zeta-potential, used for the physicochemical characterization of nanoparticles. Dynamic light scattering is used to measure nanoparticles size, but also to evaluate their stability over time in suspension, at different pH and temperature conditions. Zeta-potential is used to characterize nanoparticles surface charge, obtaining information about their stability and surface interaction with other molecules. In this review, we focus on nanoparticle characterization and application in infection, cancer and cardiovascular diseases.

## Introduction

Nanotechnology research and development have increased over the last three decades. The concern about the bioavailability and efficacy of conventional therapeutics by their suboptimal results on targeted cells and high toxicity in normal cells have lead the scientific community to reshape the vision of drug development (Geszke-Moritz and Moritz, 2016). Nanoparticles (NPs) have been developed to overcome the problems of targeting and efficiency, with reduced toxicity. In the last decade, their applicability has been focused on the biomedical and pharmaceutical fields, used as drug delivery systems, diagnostic tools, and implants (Zhang, 2015; Geszke-Moritz and Moritz, 2016; Alegret et al., 2017; Jurj et al., 2017; Ramos et al., 2017; Wong et al., 2017). Nanoparticles can be made of different materials, organic or inorganic, such as metal, polymers, carbon nanotubes, and liposomes (Liu et al., 2016). The use of nanoparticle-based drug delivery systems has increased due to their controlled release of reservoir content, leading to a decrease in undesirable side effects (Cosco et al., 2011; Mahmoodi et al., 2016; Jurj et al., 2017; Panahi et al., 2017; Singh et al., 2017). At the same time, the use of nanoparticles in drug development reduces the usage of additional components on the formulation to protect therapeutics from degradation and increase circulation time.

Nanoparticle formulation requires full characterization of its size, surface charge, shape, and distribution (Oberdörster, 2010). It is often technically challenging to obtain reproducible suspensions of nanoparticles with low polydispersion and desired shape and size. The tight control of mixing and separation of particles is crucial to obtain a homogeneous nanoparticle suspension (Cosco et al., 2015b). Usually, only a small fraction of the nanoparticles injection dose (<0.7%) reaches the target (Schmidt and Storsberg, 2015). This shows that NPs have some organism barriers to overcome, such as unspecific distribution, interstitial fluid pressure, cellular internalization, and drug efflux pumps, before achieving therapeutic effect (Park and Na, 2015).

**Figure 1:**
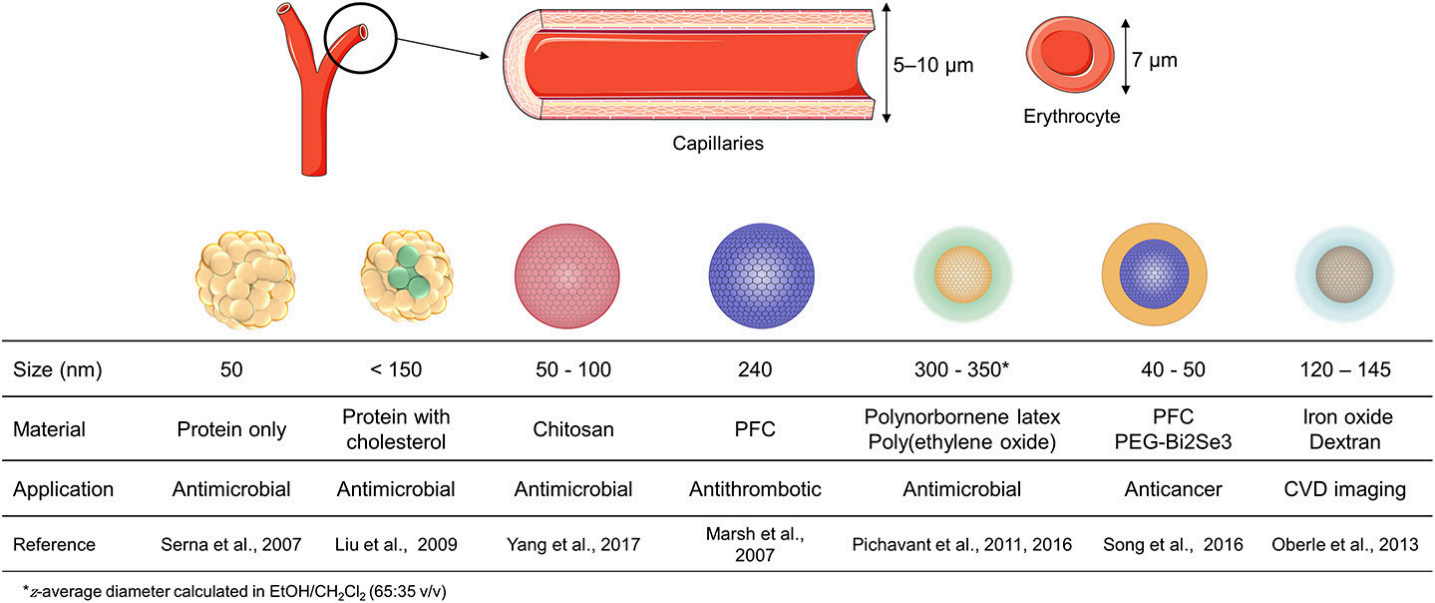
Comparison of capillaries and different nanoparticles size described in literature for different therapeutic applications. Nanoparticles were designed in terms of size and material considering the therapeutic target desired, with the size being determined by dynamic light scattering.

Nanoparticles have size-related properties influencing their mode of action and *in vivo* lifetime. The optimal size for drug delivery systems is considered to be broadly between 10 and 1000 nm (Ramos et al., 2017). Low sizes allow NPs to cross cell membranes and avoid detection by the reticuloendothelial system (RES), increasing the drug circulation lifetime (Schmidt and Storsberg, 2015; Hare et al., 2017; Jahan et al., 2017). However, they must not be too small, in order to avoid rapid distribution into lymph nodes, being eliminated by fast renal clearance. On the other hand, nanoparticles larger than 100 nm are more prone to accumulate at the site of injection or trapped by the spleen, lung, and liver macrophages (Jurj et al., 2017). In conclusion, size must be optimized taking into account the amount of cargo to be delivered and the desirable biodistribution (Figure 1).

In terms of surface charge, neutrality may lead to nanoparticle instability, with aggregation and precipitation after long-term storage. The surface charge characterization is an important parameter to measure in a NPs suspension, because the first interaction is with the body fluids before reaching a target. In physiological media, the nanoparticle is covered by plasma proteins leading to surface charge alterations and, concomitantly, changing its biological activity and affinities (Ramos et al., 2017). Positive surface charges may facilitate the binding of nanoparticles to cell membranes and might promote unspecific binding to normal tissues, promoting platelet accumulation and hemolytic events (Licciardi et al., 2016; Jahan et al., 2017; Jurj et al., 2017; Jiang et al., 2018; Peretz et al., 2018).

The unique physicochemical properties and nanoscale effects have drawn interest on nanoparticle as drug delivery systems for the treatment of diseases such as cancer, cardiovascular diseases, pathogenic infections, and diabetes. Despite the raised interest in nanoparticle development, not so many have been approved for therapeutic use (Wang et al., 2017). Here, we will focus on the light scattering approaches to characterize nanoparticle suspensions and their applicability on nanoparticle development against infectious and cardiovascular diseases.

## Light scattering techniques

### Dynamic light scattering

The detection of the light scattered from the interaction of light with matter gives information related to the physical characteristics of the sample. Typically, in light scattering experiments, a monochromatic beam is directed to the sample and then a detector records the scattered light at a certain angle. Early light scattering experiments started in late nineteenth century, with John Tyndall's research in colloidal suspensions (Tyndall, 1868). Lord Rayleigh (John William Strutt) reported another important effect of the light scattering by particles smaller than its wavelength, by explaining the blue color of the sky and the effect of the atmospheric particles (Strutt, 1871). For larger particles relative to the wavelength of light, Gustav Mie developed a theory to study the light scattering from absorbing and non-absorbing particles, considering particle shape and the difference in refractive index between particles and the medium where they are dispersed (Mie, 1908). Taking into account the differences of the light scattering at different angles of detection from large particles (Mie theory) with the more homogeneous light scattering at each angle for small particles (Rayleigh theory), we hereby use the Rayleigh particle for theoretical purposes.

In static light scattering, the intensity of the light detected is averaged over time, and from this we can obtain information about the molecular weight of the particle and its radius of gyration (*R*_*g*_). On the other hand, dynamic light scattering (DLS), by measuring over time the fluctuations of the light intensity, due to particle Brownian motion, allows to determine the diffusion coefficient (*D*), which relates to the hydrodynamic radius (*R*_*h*_) of the particle through the Stokes-Einstein equation (Pusey, 1974),

(1) $$\begin{array}{l} D=\;\frac{k_{b}T}{6\pi \eta R_{h}} \end{array}$$

where κ_*b*_ is the Boltzmann constant (1.380 × 10^−23^ kg.m^2^.s^−2^.K^−1^), *T* is the absolute temperature, and η is the viscosity of the medium.

**Figure 2:**
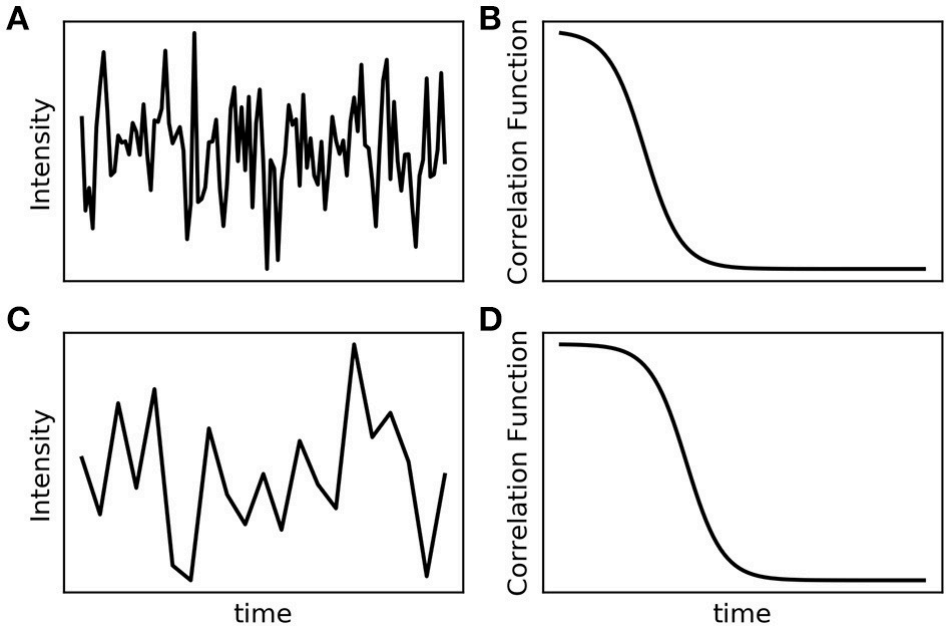
Dynamic light scattering intensity signal and correlation function for small **(A,B)** and large particles **(C,D)**. The scattering intensity signal over time is obtained directly from the particle's Brownian motion. The correlation function is obtained from the intensity fluctuation in the respective time frames. Small particles **(A,B)** diffuse faster, with the correlation decaying at early time points. Large particles **(C,D)** diffuse more slowly, which implies a longer time for the photon correlation to decay.

As it shows up in Equation (1), the particle diffusion depends on the temperature, viscosity of the media and size of the particle. DLS measures the intensity of the light scattered over time. When the intensity is correlated at several time points, in the beginning the scattered intensities are similar, losing this similarity over time due to particle's movement. Then, for small particles, the diffusion is much faster, photon correlation is lost faster and the correlation decays at early time points of the measurement (Figures 2A,B). However, as large particles diffuse more slowly, the similarity of the intensities over time persists for longer periods, leading to a longer time for the photon correlation to decay (Figures 2C,D). A digital correlation measures the intensity fluctuation and their correlation in respect to time frames (on the ns and μs timescale). The measured parameter is a normalized integration of the intensities at the beginning and a delayed time τ (Chu, 1974),

(2) $$\begin{array}{l} g_{2}\left(\tau \right)=\;\frac{\langle I\left(t\right)\;.\;I\left(t+\tau \right)\rangle }{\langle {I\left(t\right)}^{2}\rangle } \end{array}$$

However, the measurement of each particle position in the scattered volume is not possible under the experimental apparatus. For this reason, there is a measurement of the normalized electrical field generated by the volume of the particles under an incident beam (Berne and Pecora, 1976),

(3) $$\begin{array}{l} g_{1}\left(\tau \right)=\;\frac{\langle E\left(t\right)\;.\;E\left(t+\tau \right)\rangle }{\langle {E\left(t\right)}^{2}\rangle } \end{array}$$

The normalized intensity integration is correlated with the normalized electrical field measured by the Siegert relation (Siegert, 1943),

(4) $$\begin{array}{l} g_{2}\left(\tau \right)=\;B+\;\beta {\left|g_{1}\left(\tau \right)\right|}^{2} \end{array}$$

where, *B* is the baseline (~1) and β is the coherence factor, which depends on detector area, optical alignment and scattering properties of macromolecules or supramolecular aggregates. Considering a monodisperse sample, the normalized intensity integration decays exponentially and is dependent on a decay constant, Γ, for macromolecules undergoing a Brownian motion (Einstein, 1905, 1906),

(5) $$\begin{array}{l} g_{2}\left(\tau \right)=\;1+\;\beta e^{-2\Gamma \tau } \end{array}$$

where Γ is related to diffusion coefficient of the sample particles, *D*, by (Berne and Pecora, 1976),

(6) $$\begin{array}{l} \Gamma =\;Dq^{2} \end{array}$$

where *q* is the scattering vector, directly proportional to the refractive index, *n*_0_, and inversely proportional to the wavelength, λ (Harding, 1997),

(7) $$\begin{array}{l} q=\frac{4\pi n_{0}}{\lambda }\sin\left(\theta /2\right) \end{array}$$

where θ is the angle of the detector's position. However, when considering a polydisperse sample, the normalized intensity integration cannot be described by a single exponential decay (Briggs and Nicoli, 1980). Instead, there is a sum of exponential decays rates *G*(Γ) corresponding to each particle in the sample (Berne and Pecora, 1976),

(8) $$\begin{array}{l} g_{2}\left(\tau \right)=\;1+\;\beta {\left(\int_{0}^{\infty }G\left(\Gamma \right)e^{-\Gamma \tau }d\Gamma \right)}^{2} \end{array}$$

Data can be analyzed from the fitting of the correlation function. However, it is possible to distinguish two types of methods of fitting: assuming a monomodal distribution or a non-monomodal distribution. The common monomodal approach is the cumulants fitting, where a Taylor expansion with a mean decay rate is fitted to the correlation function, obtaining a mean diffusion coefficient (Koppel, 1972). From the relation of the second cumulant to the mean decay rate, it is possible to obtain the polydispersity index (PDI), informing about the monodispersity tendency of the sample. Regarding non-monomodal distribution methods, the fitting of the correlation function is based on multiple decay rates, which is more suitable for polydisperse samples. The common methodologies are non-negative least squares (NNLS), where the decay rates are constants from the list of *G*(Γ) in a determined range, but spaced linearly or logarithmically (Morrison et al., 1985). The exponential sampling uses the decay rates in a determined range but spaced exponentially. The most common methodology applied to non-monomodal distribution is the constrained regularization method for inverting data (CONTIN) (Provencher, 1982a,b). The CONTIN method is similar to NNLS, but instead of the minimization of residuals in the NNLS methodology, it works by the minimization of regularized residuals and an appropriate weighing function. For more details on the mathematical approach used in the methods, please refer to Fischer and Schmidt (2016) and Stetefeld et al. (2016).

### Zeta-potential

**Figure 3:**
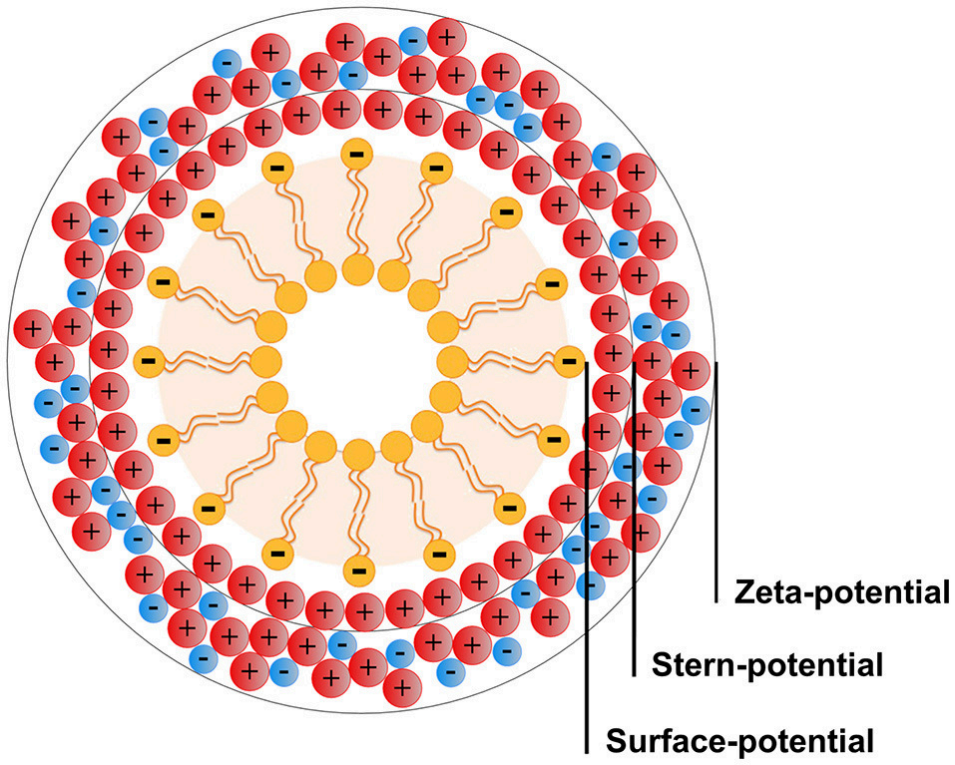
Schematic representation of the double layer that surrounds the nanoparticle in aqueous medium, considering that it has negative charge. The nanoparticle represented as example is composed by negatively charged phospholipids, implying a first layer (Stern-potential) mainly composed by positively charged counterions after application of an electric field. The second layer (zeta-potential) is a diffusive layer that consists of both counterions and ions of the same charge as the nanoparticle, which contact the organic or aqueous environment.

The zeta-potential is the potential measured at the slipping plane of a particle under an electrical field. It reflects the potential difference between the electric double layer (EDL) of electrophoretic mobile particles and the layer of dispersant around them (aqueous or organic environment) at the slipping plane (Figure 3) (Montes Ruiz-Cabello et al., 2014). The EDL surface of a particle in solution develops instantaneously and is formed of two layers. The inner layer, the so-called Stern layer, is composed of opposite charged particles tightly coupled to the core of the central particle. The second and outermost layer is a diffusive layer consisting of both opposite and same charged ions/molecules. When an electrical field is applied to the sample, the particles move to the opposite electrode. Within the diffuse layer there is a hypothetical plane that acts as the interface between the moving particles and the layer of the surrounding dispersant while in the electrical field. This plane is the characteristic slipping/shear plane and zeta-potential is the potential at this particle-fluid interface (Kaszuba et al., 2010; Bhattacharjee, 2016). The zeta-potential is measured by the electrophoretic mobility of charged particles under an applied electric field. The electrophoretic mobility (μ_*e*_) of the particles is calculated by Henry's equation (Kaszuba et al., 2010),

(9) $$\begin{array}{l} \mu_{e}=\frac{2\varepsilon_{r}\varepsilon_{0}\zeta f\left(Ka\right)}{3\eta } \end{array}$$

where ε_*r*_ is the relative permittivity/dielectric constant, ε_0_ is the permittivity of vacuum, ζ is the zeta-potential value, *f* (*Ka*) is the Henry's or Helmholtz-Smoluchowski function, and η is the viscosity at the experimental temperature. Depending on the solvent where the particles are dispersed, the value of *f* (*Ka*) is assumed to be 1 or 1.5, for organic medium or aqueous medium, respectively (Domingues et al., 2008).

## Nanoparticles in therapeutics

Nanoparticles are widely used in biomedical sciences for different therapies, due to their high biocompatibility and chemical stability, either by direct activity or by encapsulating poorly soluble drugs/surface incorporation (Arakha et al., 2015; Elzoghby et al., 2015). Among the most notorious examples are magnetic nanoparticles, with a metal core of Zn, Ni, Cu, Ag, or Au, synthetically obtained or naturally isolated (Bilal et al., 2017; El-Batal et al., 2018). Some of these were shown to have antimicrobial activity, and were considered perfect candidates for magnetic resonance imaging techniques, presenting a dual activity: therapeutic and diagnostic (Niemirowicz et al., 2015; Dinali et al., 2017). Their use in bandages, implants or prostheses is already becoming common, but overproduction of reactive oxygen species (ROS) in long-term usage has raised concerns regarding the toxicity of magnetic NPs (Bilberg et al., 2012; Casciaro et al., 2017). Different authors have explored this issue, even in polymeric-coated magnetic NPs, which were consider less toxic than the uncoated, but high dosages during a larger period of time increase cytotoxic and genotoxic effects on macrophages (Jena et al., 2012; Mohanty et al., 2012). With nanoparticles activity being dependent of their physicochemical properties, namely size, shape, and surface, their toxicity toward cells is also dependent of these properties (Bera et al., 2014; Sun et al., 2014; Rajchakit and Sarojini, 2017). A strategy followed to deal with these problems has been the development of different types of nanoparticles, including polymeric nanoparticles, micelles, or liposomes, with the advantage of being possible to shape their properties to increase the efficacy in targeting or drug delivering (Xie et al., 2014; Bilal et al., 2017; Solairaj et al., 2017). With the objective of reducing toxicity without reducing NP activity, another adopted strategy was the incorporation or surface derivatization with different ligands, such as antibodies, small organic molecules, or proteins/peptides (Chen et al., 2012; Gao et al., 2017). This last hypothesis was shown to reduce toxicity, improving peptide properties/activity, and enhancing solubility, leading to a general improvement of the pharmacokinetic profile and therapeutic index (Molinaro et al., 2013; Gao et al., 2014, 2017; Cosco et al., 2015a; Libralato et al., 2017). As a matter of fact, different proteins have already been tested for different activities, including albumin, casein or elastin-like polypeptides, exploring either an active targeting (direct activity on target cells) or a passive targeting (prolonged blood circulation and activity) (Sneharani et al., 2010; Zhao et al., 2010; Bachar et al., 2012; Kratz, 2014; MacEwan and Chilkoti, 2014). With different possibilities of surface modification, these protein nanoparticles rapidly evolved to peptide nanoparticles, due to an easier manufacture process and reduced production costs (Elzoghby et al., 2015).

Peptide therapeutics is a field that is fast growing since the beginning of the century, with a large number of scientific papers exploring their potential use in healthcare (Albericio and Kruger, 2012). Moreover, with resistance increase in different pathologies, including infectious diseases and cancer, the urgency for new alternatives has promoted a significant number of studies aiming at improving the efficacy of peptides as drugs or applied in diagnostic techniques (Hamilton et al., 2015; Gomes et al., 2018). Several *in vitro* and *in vivo* studies have been published focusing on the efficiency of particular peptide classes, namely antimicrobial and anticancer peptides (AMPs and ACPs, respectively), due to their promising applications as drugs in the market (Hancock et al., 2016; Felício et al., 2017). Even so, downsides of their use have been pointed out, including low enzymatic stability, low permeability across biological barriers, low solubility, rapid metabolic excretion, and high toxicity (Tam et al., 2002; Rajchakit and Sarojini, 2017; Serna et al., 2017). Strategies to overcome these peptide therapeutic applicability problems include *in silico* structure design (considering their sequence, using natural and non-natural amino acids), peptidomimetics, lipidation and, naturally, nanoparticle conjugation, which will be further explored (Rajchakit and Sarojini, 2017; Primavera et al., 2018).

### Nanoparticles with antimicrobial activity

As mentioned above, an increase in multiresistant pathogens (bacteria, fungi, and viruses) has been reported on the last decades, with several reasons already explored being held responsible for this (Dickey et al., 2017; Llewelyn et al., 2017). The World Health Organization has inclusively pointed out different bacteria strains where researchers should focus on, due to the high incidence of resistance in patients (World Health Organization, 2015). AMPs are considered one of the major promises to overcome this growing public healthcare problem. Due to this, studies on their isolation, purification, design, and applicability, both *in vitro* and *in vivo*, have increased in recent years (Dias et al., 2017; Unubol et al., 2017). These peptides are usually characterized by a short amino acid sequence (less than 50 amino acid residues), high amphipathic and hydrophobic content, and a positive net charge (de la Fuente-Núñez et al., 2017; Haney et al., 2017). Their mechanisms of action, frequently at the membrane level, are not well-defined, but it is clear that their physicochemical properties are essential for the peptide-membrane interaction (Neelay et al., 2017). Initially, they were thought to target specifically different pathogens but, nowadays, it is clear that their action is more complex than that, participating in the recruitment of immune cells to the site of infection, or modulating the immune response by promoting pathogen cell death (Hancock et al., 2016). Also, they have a broad-spectrum activity (Vigant et al., 2015), being active toward bacteria (including biofilms), fungi or viruses, with properties of the target membrane driving the interactions (Ribeiro et al., 2016). Even so, their limitations became also notorious in a large number of studies, limiting their potential as therapeutic molecules (Gomes et al., 2018).

At the same time, nanotechnology (particularly using nanoparticles) has also focused its research in these applications, having reached a higher success in clinical applications. With the advantage of the possible use of different metals, magnetic nanoparticles with intrinsic antimicrobial activity were developed and medically applied (Bilal et al., 2017; Dinali et al., 2017; Pham et al., 2018). The fact that these NPs have in their core a metal predisposing to electrostatic interactions, promotes their attachment to bacterial membranes, leading to the loss of integrity and bacteria cell death (Fang et al., 2015; Bilal et al., 2017). A high number of systems have been tested with reported activity toward pathogens, using different antibiotic molecules conjugated either on the surface or by encapsulation (Park et al., 2011; Gaspar et al., 2016; Morales et al., 2017). An important example is silver nanoparticles (AgNPs) conjugated with polymixin B or gold nanoparticles (AuNPs) conjugated with vancomycin, both showing a synergistic effect, with improved activity (Fayaz et al., 2011; Park et al., 2011). Metal nanoparticles chosen for antibiotic conjugation include titanium, zinc or cooper, and as for antibiotic molecules, gentamicin, streptomycin, cecropin-melittin, among others, have shown improved activity (Gu et al., 2003; Birla et al., 2009; Allahverdiyev et al., 2011; Lai et al., 2015). However, as already stated, the use of metals for nanoparticle development raised some doubts due to inherent toxicity toward healthy cells, forcing researchers to find alternatives. An example was testing NPs for local/topic applications, lowering the dosage amount and toxicity effects (Gao et al., 2014; Arakha et al., 2015; de Oliveira et al., 2017). In a pH-sensitive system, Pichavant et al. developed antibiotic (gentamicin sulfate and/or vancomycin) functionalized nanoparticles that were covalently grafted into titanium surfaces (Pichavant et al., 2016). The nanoparticle characterization was achieved using nuclear magnetic resonance (NMR) and dynamic light scattering measurements, confirming their size and stability in different media (Pichavant et al., 2011, 2012). Besides the enhanced antimicrobial activity, these NPs also presented other advantages, such as the possibility to be used in other surfaces and promoting an increase in the target tissue/cell drug density (Pichavant et al., 2011, 2012). Another study, by Di Francesco *et al*. showed the advantages of using pH-sensitive nanoparticles as a fusogenic drug delivery system (Di Francesco et al., 2017). The nanoparticles were formulated according to their target cells, and physicochemical properties were measured by DLS and fluorescence spectroscopy.

The application of nanolipid systems (like liposomes or micelles) or polymeric NPs (chitosan based or conjugated with polyethylene glycol, PEG) had special success (Hann and Prentice, 2001; Allen and Cullis, 2013; Cosco et al., 2014; Paolino et al., 2017). These NPs have the advantage of being more biocompatible, with the effects toward healthy cells being reduced and having an improved targeted-oriented activity (Solairaj et al., 2017). Water et al. developed poly(lactic-co-glycolic acid) nanoparticles (PLGaNPs) that were used as drug delivery system for plectasin, an antibiotic specific for airway *Staphylococcus aureus* infection (Water et al., 2015). They used DLS and zeta-potential measurements to assure that plectasin was efficiently loaded on the NP. Other authors used chitosan-sodium phytate NPs and tested them against Gram-negative and Gram-positive bacteria, showing a high antimicrobial activity, with the advantage of these NPs could also be used for drug delivery, combining their efficacy with an antibiotic (Yang et al., 2017). In order to identify the optimal chitosan/sodium phytate ratio for their activity, DLS and zeta-potential measurements were performed to determine the NPs size, surface charge, and stability at different pH values. As for liposomes, other authors have developed lipid NPs composed of phosphatidylcholine (zwitterionic phospholipid) and phosphatidylserine (negatively charged phospholipid), intercalated with Pluronic-P85 (HLB 16), a polymer that favors the uptake of the NP (Fidler, 1988; Zhang et al., 1998). By incorporating gentamycin in their core, this system was tested for drug delivery, with a high efficiency rate (Xie et al., 2014).

Despite all the strategies studied along the years, one has gained special attention nowadays, when conventional therapeutic molecules are facing a new resistance paradigm. This strategy consists in the combination of nanoparticles (liposomes, polymeric, or metallic) with antimicrobial peptides, either for peptide delivery or for a direct action toward the target cells (Niemirowicz et al., 2015; Water et al., 2015). The objective was to overcome the limitations on AMPs application, but, later on, it was stated that the nature of nanoparticle-AMP interaction is essential for the system activity (Pal et al., 2016). Actually, weaker interactions between the AMP and the nanoparticle promote a decrease in NP toxicity and, at the same time, increase AMP activity, because it allows the peptide to adopt favorable structure and/or charge properties essential for the interaction with biomembranes (Liu et al., 2013; Rajchakit and Sarojini, 2017). These AMP-NP complexes also allow a higher concentration of the drug in the site of action, with a selective activity, including a differential interaction between the complex and the outer and inner-membranes of the target bacterial pathogens, implying a drug-delivery and direct activity system (Park et al., 2017; Rajchakit and Sarojini, 2017).

Different AMP-NP complexes have been tested throughout the years, trying to establish one with high activity toward the target pathogens, without having a significant toxicity for the other cells, a common flaw for AMPs and metal NPs (Galdiero and Gomes, 2017). Different metals were tested, as already described, such as iron oxide, coupled with LL-37, a natural host-defense peptide with antimicrobial activity (Niemirowicz et al., 2015). Other examples include silver nanoparticles surrounded by AMPs, or gold NPs with bactenecin molecules on their surface (Allahverdiyev et al., 2011; Golubeva et al., 2011). All these systems were shown to have less toxicity and higher efficiency, including against clinical isolated multiresistant pathogens, but their pharmacokinetic and pharmacodynamic profiles still need to be improved (Ruden et al., 2009). Considering this scenario, methods to improve these properties were designed, including the use of natural isolated nanoparticles from biomass (Mohanty et al., 2013). Their biogenic AgNPs combined with two different AMPs (NK-2 and LLKK-18), characterized by DLS and zeta-potential, were shown to have synergistic effect and improved applicability in clinical scenarios (Mohanty et al., 2013). In another study, polymeric nanoparticles (chitosan-alginate polyelectrolyte complex NPs) combined with pexiganan (a synthetic AMP) had an improved profile for therapeutic application (Zhang et al., 2015). Another strategy tested was the use of PEG: nanoparticle surface was covered with PEG and AMPs, increasing biocompatibility and antimicrobial properties (Pal et al., 2016; Casciaro et al., 2017). Zeta-potential was used to confirm that the peptide was attached to the NP surface after coupling synthesis, with an overall charge increase after interaction with positive peptides such as AMPs.

Besides antimicrobial peptides, nanoparticles can also be combined with cell-penetrating peptides (CPPs) (Guidotti et al., 2017). There is not a rigid boundary between these two classes of peptides, with reported AMPs having a CPP function, as well as CPPs with described antimicrobial activity, besides the capacity to deliver cargo into different cells (Bahnsen et al., 2015; Kristensen et al., 2016). One example is the combination of micelles with TAT, a HIV-derived CPP with antimicrobial activity, conjugated with cholesterol, a spacer and six arginine residues (Liu et al., 2009). These self-assembly micelles, besides enhanced activity and low toxicity, were able to cross the blood brain barrier, which introduced a great advantage for brain infection diseases (Liu et al., 2009). On another study from the same authors, they used the same CPP, with a spacer of three glycine and six arginine residues, but conjugated to colloid AgNPs surface (Liu et al., 2013). Improved antimicrobial activity and reduced hemolysis were observed. In both cases, DLS and zeta-potential were essential to characterize the NPs, regarding size and surface charge, but also to assess colloidal stability (Liu et al., 2009, 2013).

It is important to refer that these complexes of AMPs/CPPs-NPs have high potential for the treatment of bacterial infection, including those leading to biofilm formation (Ribeiro et al., 2016). Biofilms are complex pathogen aggregates, encased in a matrix composed of extracellular polymeric substances (EPS), that normally tend to form when bacteria faces stress adaptation (Flemming et al., 2016). Due to this matrix, AMPs efficient against planktonic (free) bacteria can be ineffective against biofilms (Batoni et al., 2016). Nanoparticles by themselves have small size, with an enormous surface area and easy penetrability properties, including on biofilms. These properties and association with AMP introduce advantages to tackle biofilm infection, and should be considered in future works (Qayyum and Khan, 2016).

The use of peptides in nanotechnology has been largely increasing, as described above. At this level, other structures with promising results are self-assembling peptide NPs, which are formed by small peptides that self-aggregate, forming clusters, or oligomers (Serna et al., 2017). The idea came from dendrimeric peptides, small nanosystems with a size range from 2 to 50 nm, with great advantages in terms of biocompatibility, structural/functional versatility and drug delivery efficiency (Tam, 1988; Tam et al., 2002; Serna et al., 2017). These systems are characterized by a hyper-branched and almost perfect geometrical 3D architecture, that grow from the core into a globular shape, with reported activity against infectious pathogens and cancer cells (Tam et al., 2002; Ionov et al., 2013; García-Gallego et al., 2017). Examples of systems already studied are diverse, with each author exploring different mechanisms to promote the assembling or activity toward the target cells. They include AMPs conjugated to the N-terminal of histidine-tagged proteins, forming oligomers with antimicrobial activity (Serna et al., 2017). As the synthesis of self-assembly NPs starts with small aggregates, DLS was used to determine the evolution of the size distribution, confirming the oligomers formation (Serna et al., 2017). Lipidation of AMPs, besides the increased activity already explored, can also promote the formation of dendrimeric peptide NPs (Siriwardena et al., 2018). Using parental systems, these authors developed a new one, with higher antimicrobial activity and pro-angiogenic properties in biological burn-wound bandages, named TNS18 (Siriwardena et al., 2018). Finally, other authors recently focused in self-assembling peptide nanoparticles that only act on the target cell after activation, using for that specific characteristics of the target tissue, such as overexpressed membrane proteins or enriched proteases concentration (Yu et al., 2018; Zhang et al., 2018). This field is now expanding and, therefore, more research is needed to understand how this strategy can benefit current therapies relative to other systems that are easier to manipulate.

### Nanoparticles with anticancer activity

Therapies to deal with cancer have evolved in response to the human need, but resistance to therapy is a public health concern (Arnold et al., 2015). Nanotechnology has for long tried to fight this burden, by improving the pharmacokinetic and pharmacodynamics of the chemotherapeutic agents that target solid tumors. For that, drug encapsulation was studied and tested *in vivo*, with the first molecules being FDA approved in the middle of the 1990s, namely Doxil and DaunoXome (Eertwegh et al., 2006). Both therapeutics consist of liposomes with encapsulated drugs, doxorubicin (DOX) and a mixture of anthracycline and daunorubicin, respectively (Eertwegh et al., 2006; Allen and Cullis, 2013). Cancer drugs face diverse challenges, creating the need of developing new drugs according to the type of target: solid tumors or circulating cancer cells (Pearce et al., 2012). For solid tumors, evading the mononuclear phagocyte system (MPS) and remaining in the tumor tissue is essential for drug efficacy, while for circulating cancer cells there is the need for the drug to be internalized to ensure its action at the target site (Stylianopoulos and Jain, 2015).

Considering the current scenario, different strategies have been followed trying to overcome these limitations. Metal nanoparticles with gold or silver core have been tested and showed to have natural anticancer activity, either *in vitro* or *in vivo* against tumors and cancer cells (Shanmugasundaram et al., 2017; Shmarakov et al., 2017). Following the improvements on the development of nanoparticles, combinations of copper and chitosan were also tested, with observable anticancer activity and less toxic effects (Solairaj et al., 2017). DLS was used here not only as a mere characterization technique, but as a tool to identify metal structures with higher colloidal stability and better size distribution (Shmarakov et al., 2017). Di Francesco *et al*., using non-ionic surfactant vesicles (NSVs) loaded with DOX, developed nanosystems with different ratios of Tween21/Tween80, promoting a pH-responsive approach with anticancer properties (Di Francesco et al., 2017). These NSVs showed a fusogenic behavior and an increased targeting efficiency, which translated in higher anticancer activity. Nanoparticles with direct activity can also be used as drug carriers, as mentioned before. Zakerzadeh *et al*. designed silica NPs with encapsulated tetrazole, a cyclic/aromatic molecule with antimicrobial, antifungal and anticancer activity (Zakerzadeh et al., 2017).

Despite previous advances, improvements in the targeting were still necessary. As in infection therapies, also here the use of peptides was considered, either to increase activity or to promote specific targeting to cancer cells and solid tumors (Pearce et al., 2012). As an example, Chang *et al*. designed NPs that were able to bind to the tumor mass (oral, breast, lung, colon, or pancreatic tumors) by coating them with the small antimicrobial peptides PIVO-8 and PIVO-24 (also acting at the vascularization process), which are significantly increased around tumors (Chang et al., 2009). To confirm NP coating with both peptides, authors used DLS, evaluating afterwards the differences in activity (Lee et al., 2004). Also targeting tumors, iron oxide NPs coated with an heptapeptide that recognize fibrin-fibronectin complexes or chitosan NPs with antiangiogenic peptide endostatin (ES) improved anticancer activity by targeting the vascularization of the tumor (Agemy et al., 2010; Xie et al., 2017). Coating nanoparticle surfaces with two or more different peptides was also reported (Colombo et al., 2002; Marchiò et al., 2004). Even so, ideally, anticancer therapies would be able to eliminate tumors and malignant cancer cells, including those that are no longer associated with the main tumor, without toxicity toward healthy cells. NPs that act as drug carriers (for drugs like doxorubicin, 5-fluororacil or cisplatin), with good pharmacokinetic and pharmacodynamic profiles (using PEG on their surface or polymer NPs), specific (by using small peptides) and with enhanced cellular uptake would be the desired candidates (Safra, 2003; Paolino et al., 2013; Ribeiro et al., 2016; Gomes et al., 2018). For this system, the missing point is the enhanced uptake, which was possible with the attachment of CPPs to the nanoparticle surface, besides the AMPs necessary for their activity. Authors tested the use of TAT, the HIV-1 derived CPP, by coupling it to NPs with PEG on their surface, and demonstrated the improved cellular uptake (Kuai et al., 2010, 2011). Besides the CPP, these authors also tested the possible applicability of different PEG molecules, due to their concern for increasing NPs distribution near the tumor, but loss of internalization ability (Kuai et al., 2010). Using DLS and zeta-potential measurements, Kuai *et al*. studied the optimal proportion of cleavable PEG to maintain their accessibility and activity. Takara et al. also tested the incorporation of a CPP (STR-R8) on the nanoparticle surface, that was already coated with NGR motif peptides (recognizes CD13 presence in endothelial tumor cells) and PEG, showing that a synergic effect between all the molecules incorporated occurred (Takara et al., 2010). For that, DLS and zeta-potential were used to evaluate the best CPP amino acid residue to use for the anchoring, considering that size should be stable, and that surface charge is essential for NP targeting membrane interaction. Recently, Xia *et al*. further increased the complexity with a high efficiency construct: using selenium NPs, which have advantages in terms of dosage, biocompatibility, toxicity, and drug delivery, they coated them with an anticancer peptide (RGDFC heptapeptide) and incorporated DOX and siRNA (anti-Nanog, a human homeobox protein that is essential for cancer cell proliferation) (Xia et al., 2018). This SeNPs@DOX/siRNA system showed to be very effective on the targeting and treatment of cancer, presenting a new hypothesis as synergistic system. Nevertheless, regarding cancer therapies, there is a lot to improve in terms of targeting and efficiency of cancer eradication *in vivo*, because most of the systems tested *in vitro* have been failing on clinical trials (Pearce et al., 2012).

### Nanoparticles in cardiovascular diseases

Although areas like cancer and antimicrobial resistance draw most of the attention from the public and scientific community, cardiovascular diseases (CVD) are the major epidemic of the modern era, claiming a higher number of deaths than cancer, malaria, AIDS, or tuberculosis. Indeed, CVD remains the most common cause of death worldwide (Park et al., 2008). Just in Europe, CVD are responsible for 45% of all deaths, reaching 4 million deaths per year (Townsend et al., 2015). Coronary heart disease is the most common single cause of death, resulting in 19% of deaths in men and 20% of deaths in women, much higher than breast cancer in women (2%) and lung cancer in men (6%) (Townsend et al., 2015). Most conventional therapeutics and clinical approaches are outdated, and researchers are putting their efforts into fast employing all the potential of “nano” in the CVD management, approaching strategies for both imaging and treatment of these conditions.

#### Developing new agents for CVD imaging

Conventional medical tools still fail on the detection of atherosclerotic lesions and plaque rupture, while interventions with a catheter ultrasound or magnetic resonance imaging (MRI) give purely morphological information, without stating the progression of inflammation and the occurring of functional changes (Park et al., 2008). New imaging techniques and agents are in high demand. Contrast agents incorporating nanoparticles and peptides have significantly evolved and are now capable of detecting and quantifying microthrombus. Nonetheless, they mainly consist of hard particles, which present excretion difficulties and slow or inexistent metabolization. The tendency is to look for more compliant particles, like self-assembling and small molecules, capable of flowing through the microvasculature of clearance organs (Pan et al., 2009), with low toxicity, good biodegradability, and biocompatibility (Park et al., 2008). There have been advances in the development of fibrin-specific manganese nanocolloids, that successfully reach the low nanomolar range of detection and present a high relaxitivity (Pan et al., 2009). These results are directly compared to the micromolar range only of the mostly used gadolinium-based agents.

**Figure 4:**
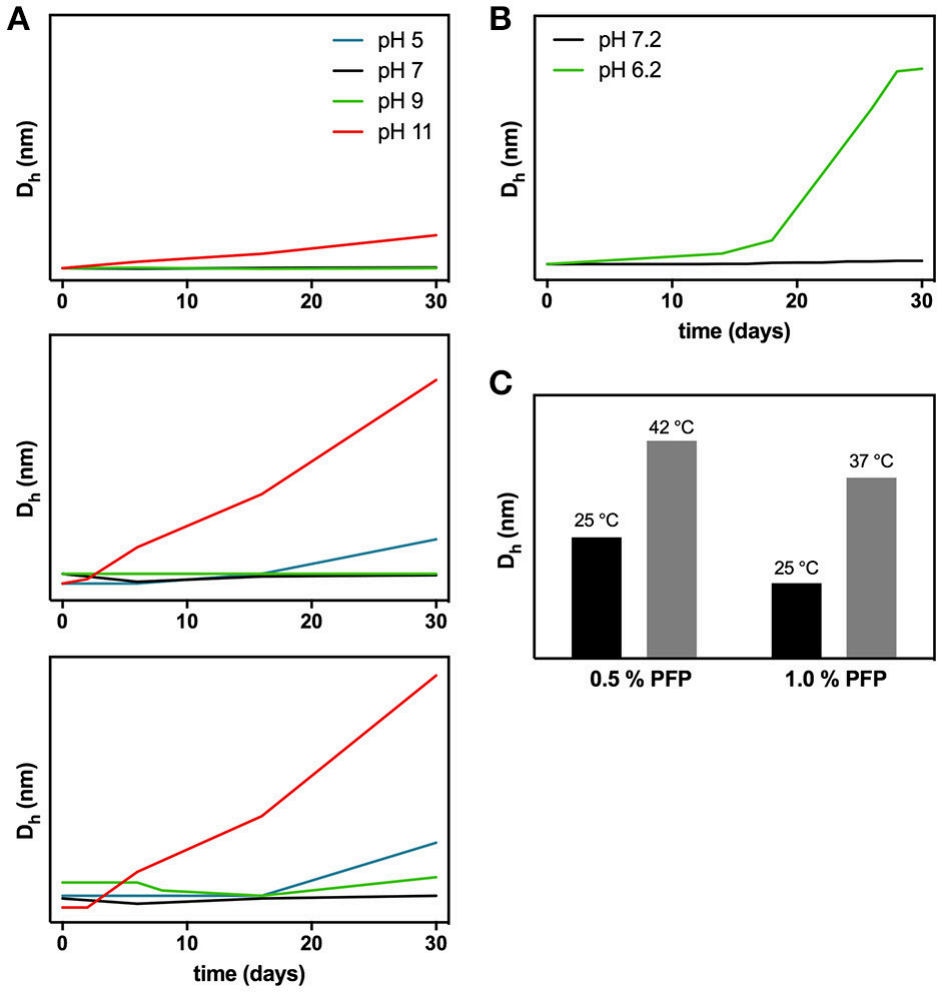
Example of dynamic light scattering applications to study nanoparticle stability at different pH values **(A,B)** and temperatures **(C)**. **(A)** Iron nanoparticles (FeNPs) aggregation stability studied over time at four different pH values. At the top, FeNPs without surface polymer; in the middle FeNPs, coupled with PEG2000 and at the bottom FeNPs couple with PEG5000. Adapted with permission from Park et al. (2012). Copyright 2018 American Chemical Society. **(B)** SPIONs aggregation stability studied over time at two pH values. Adapted from Oberle and Lüdtke-Buzug (2013). **(C)** Perfluoropentane (PFP) micelles size stability studied at different temperatures. Micelles were prepared with different percentages of PFP (Rapoport et al., 2007).

In recent years, some approaches previously used mainly for oncology imaging have been adapted to cardiovascular imaging, as it is the case of iron oxide nanoparticles, especially in the form of ultra-small supermagnetic iron oxide (USPIO) nanoparticles (<50 nm) (Ploussi et al., 2015). Early use of these NPs for medical imaging was described as a solution for the limiting factor in MRI, the background signal produced by the host tissue, but they can also be used for magnetic particle imaging (MPI), being capable of providing a higher sensitivity and a better spatial resolution (Gleich and Weizenecker, 2005). Due to the high interest in these particles, several variations of superparamagnetic iron oxide nanoparticles (SPIONs) can be found, as well as the characterization of their behavior in different situations. Park *et al*. have subjected three formulations of SPIONs to pH variations (5, 7, 9, and 11) and time progression (30 days) (Park et al., 2012). By light scattering analysis at pH 11, a significant increase in hydrodynamic diameter was observed, leading to the conclusion that nanoparticle aggregation is occurring, especially when PEG was one of the components (Figure 4A). Under further analysis, authors concluded that the PEG coating was desorbed from the surface, leading to an unstable NP suspension and triggering aggregation. At pH 7, there were no alterations in measured sizes for the particle.

SPIONs can have multiple coating options. Thus, authors can play with either PEG or other biocompatible molecules, like chitosan. Szpak *et al*. studied the stability of iron oxide nanoparticles coated with a thin layer of charged chitosan derivatives (Szpak et al., 2013). Performing DLS measurements, they concluded that the diameter for the negatively charged NPs was slightly smaller, indicating an effect of the charge in the behavior of its milieu. Further characterization of the coating charge was performed by zeta-potential measurements. Authors emphasized that, for biological applications, SPIONs must be resistant to adsorption of biomacromolecules and that chitosan might be the ideal candidate for facilitating several degrees of physical properties manipulation, such as the tailoring of surface charge. Another study has analyzed the stability of SPIONs at 37°C, but this time with a dextran coating (Oberle and Lüdtke-Buzug, 2013). The nanoparticles were stable at pH 7.2 for as long as 6 weeks, while at pH 6.2 the hydrodynamic diameter strongly increased, denoting particle aggregation, also visible by precipitation (Figure 4B). This strongly suggests that dextran is not only a biocompatible polymer, but also an excellent solution to keep a SPION formulation stable at physiological conditions.

Iron oxide agents have been tested for the detection of abdominal aortic aneurism (Richards et al., 2011; Sadat et al., 2011), atherosclerotic plaques (Schmitz et al., 2001; Kooi et al., 2003), and acute myocardial infarction (Alam et al., 2012; Yilmaz et al., 2012, 2013a,b). Tang *et al*. also extensively studied the use of ferumoxtran-10 for imaging carotid plaques (Tang et al., 2009a,b), carotid stenosis (Tang et al., 2006) and carotid atheromas (Tang et al., 2007, 2008). Moreover, several of these tests are basically giving a new use for ferucarbotran (Resovist), an agent firstly used to detect either benign or malign hepatic lesions (Namkung et al., 2007). The particles usually have a core of magnetite (Fe_3_O_4_)/maghemite (γFe_2_O_3_) coated with carboxydextran, and an overall hydrodynamic diameter of 62 nm (Reimer et al., 1995). By 2015, only Resovist was available in very limited countries, with other agents being stopped for further development. This is the case of ferumoxtran-10 (Sinerem), also widely tested in the cited studies for cardiovascular conditions, although initially developed for lymph node imaging. Here, the core is a crystalline inverse spinel structure of magnetite, coated with dextran, with 20 nm diameter (Shen et al., 1993).

At the same time, the field is also actively looking for new disease biomarkers and intensively exploring agents involved in the inflammatory response in CVD. In an effort to provide better quantitative macrophage imaging in vascular tissue, Keliher *et al*. developed a class of modified polyglucose nanoparticles, with a size below the limit for renal excretion (Keliher et al., 2017). When macrophages fail in removing cholesterol deposits from the arterial wall, an inflammatory response is triggered, with recruitment of more cells, which enhances inflammation and compromises blood flow and tissue integrity. Animal studies succeeded in detecting atherosclerotic regions with practically no interaction with other lymphocytes. The same happened when using mice with permanent coronary ligation (Keliher et al., 2017).

With CVD prevention being the focus of significant attention from the scientific community, and studies pointing to new disease biomarkers steadily reaching publications (Hijazi et al., 2016; Walters et al., 2016), some authors explored CVD relationship with other medical conditions (Gerdes et al., 2014; Pavo et al., 2015). Following the work on screening for a peptide to bind to atherosclerotic plaques (Hong et al., 2008), other authors have carried out its incorporation as a targeting moiety in chitosan nanoparticles (Park et al., 2008). After working on hydrophobic modified glycol chitosan (HGC) nanoparticles as cancer imaging probes (Park et al., 2007) and for other therapeutic purposes (Kwon et al., 2003; Park et al., 2004; Kim et al., 2006), the team was able to conjugate the peptide on the NP surface and detect the selective binding to atherosclerotic plaques *in vivo*, by adhering to the IL-4 receptor on endothelial cells, macrophages and smooth muscle cells (Park et al., 2008). The authors highlighted that these 270 nm self-assembled nanoparticles have a long residence time even in flow conditions. In fact, the fluorescence in the aortic arch of the *Ldlr*^−/−^ mice exhibited a more prominent fluorescence signal than the aortic arch of healthy mice, even after 6 h from intravenous administration.

#### Drug delivering nanoparticles for CVD treatment

The delivery of a therapeutic drug through a nanoparticle vehicle allows high drug concentrations in the intended local environments, while the total drug concentration and side effects are significantly reduced (Chen et al., 2015). In CVD, the introduction of these therapeutic agents can be done either with surgical intervention or through systemic administration. In cases of coronary artery disease, a common approach is a percutaneous coronary intervention. This procedure is performed under local anesthesia and involves the insertion of a guidewire into the aorta, to then pass other therapeutic tools, such as inflatable balloons, stents, and catheters (Chen et al., 2015). An usual side effect is restenosis, which is a narrowing of the artery, either by remodeling and recoiling of the vessel lumen, or by proliferation of smooth muscle cells in response to the injury caused by the inserted devices (Cyrus et al., 2008). The insertion of a stent is indicated to prevent the situation, but it may itself be a trigger to a proliferative response, and also a vehicle for cell migration, decreasing the internal diameter of the treated vessel. For this reason, it is important to develop modified coatings. The real advantages of using nanoparticle infused polymers are still under evaluation, with some studies concluding that the use of drug-eluting stents has a risk for thrombosis at least as great as with bare-metal stents, showing no significant effect in long-term survival and a reduction in the need for re-intervention (Kastrati et al., 2007). Other authors have shown that although successfully preventing restenosis, drug-eluting stents have a major impact delaying endothelial healing (Cyrus et al., 2008). That occurs because the coatings are made using cytostatic agents, like sirolimus and paclitaxel (Brito and Amiji, 2007). Nonetheless, despite growing expertise in manipulating surfaces, structures and materials containing nanoparticles, it is also important to keep in mind that the nanotopography also plays a role in promoting cell mobility, adhesion, and differentiation (González-Béjar et al., 2016).

The increased variety of innovative materials for stent manufacture as also raised some awareness to determine, not only which have a better drug releasing performance, but also which are the safest to use in *in vivo* magnetic particle imaging. Wegner et al. explored the heating patterns of stents made from stainless steel, nitinol, platinum-chromium, and cobalt-chromium, from several diameters and lengths (Wegner et al., 2018). The study concluded that temperature increase is a real concern in larger stents, with diameter playing a leading role. In addition, the authors suggest that a combination of geometries, and conductive and non-conductive materials would most probably prove to be the best approach for stent design.

On another approach, we can also find the direct targeting of blood clots by vehiculation of thrombolytic agents. Recombinant tissue plasminogen activator (tPA) or streptokinase are administered in case of ischemic events, despite frequent complications, such as severe hemorrhage. Both agents act by activating plasmin, which will then lyse the fibrin from the clot structure. By directing the action to the specific clot site, it is possible to diminish the administered doses, maintaining or even improving the outcome, and avoiding a systemic effect that leads to the unwanted symptoms (Elbayoumi and Torchilin, 2008; Kim et al., 2009; Koudelka et al., 2016).

Perfluorocarbons have long been a target of scientific interest for their properties of transport and delivery, being firstly studied for their ability to dissolve oxygen, and later modified with several less toxic variations to produce emulsions, as a substitute of blood components (Clark and Gollan, 1966; Mitsuno et al., 1984; Riess, 1984). Nowadays, perfluorocarbons are still being explored for their drug carrying properties, with applications in both prevention and treatment of medical conditions (Chang et al., 1988; Schad and Hynynen, 2010; Song et al., 2016; Vemuri et al., 2016). Rapoport *et al.*, for example, have already studied the influence of temperature on the stability of PEGylated perfluorochemical formulations (Rapoport et al., 2007). Following size evolution up to 42°C, by DLS, maintaining temperature for 5 min and cooling the sample before size measurements, the authors were able to observe the transformation of the nanodroplets in microbubbles within the prepared formulations (Figure 4C). Perfluorocarbon nanoparticles were also derivatized for fibrin targeting in blood clot (Lanza et al., 1996). Authors used a biotinylated form of the emulsion to target the NPs to thrombin. As a way of ensuring the presence of functional biotin at the surface, they performed an avidin titration while measuring particle size by DLS. The method revealed a steady increase in size with increasing concentrations of avidin, which demonstrates the successful functionalization of the emulsion. In another application, *in vitro* studies have demonstrated the lytic activity of perfluorocarbon NP formulations directed to clot dissolution. Marsh *et al*. successfully conjugated streptokinase on the surface of NPs made mainly of fluorooctylbromide, egg yolk lecithin, cholesterol and MPB-PE (1,2-dioleoyl-*sn*-glycero-3-phosphoethanolamine-N-[4-(p-maleimidophenyl)butyramide]) for streptokinase conjugation (Marsh et al., 2007). The *in vitro* assays showed an almost complete lysis of the human plasma clots in less than 60 min. In fact, Banai et al. demonstrated that the administration of a nanoencapsulated drug, in this case tyrphostin AGL-2043, can even be more clinically interesting than its surface adsorbed or even free form, in reducing in-stent neointimal formation (Banai et al., 2005). The *in vitro* characterization of the particles used by Banai *et al*. on their studies was previously studied by other authors (Chorny et al., 2002). These authors focused on describing a modified nanoprecipitation method for optimization of the particles' size, drug recovery yield, and release kinetics. NPs intended for intravascular delivery must be developed under optimal conditions. The authors used DLS measurements to evaluate the influence of the polymer poly(D,L-lactide) (PLA) concentration and ethanol presence in production varying sizes of particles. The size of the NPs increased with the increase of the PLA concentration used and decreased by increasing the concentration of ethanol. Ethanol impairs the solubility of PLA, decreasing the time for precipitation when in contact with an aqueous phase, producing smaller droplets. Authors also stress the importance of size in the release of the therapeutic agent, with their data showing that smaller particles had higher release rates, as a result of a greater surface area exposed to the medium (Chorny et al., 2002).

#### Combining imaging and first-line treatment—theranostics

Theranostics is a field of individualized medicine that arises from combining diagnostics and therapy, being possible due to the capacity of nanoplatforms to carry cargo and target a specific agent. Being a major aim on the area of cancer research, on the specific context of cardiovascular diseases the ultimate goal is to non-invasively define atherosclerotic burden, to deliver effective targeted drug at a fraction of previous levels, and to quantify local response to treatment (Winter et al., 2006). Although still far from meeting clinical standards, this is fast progressing, with *in vivo* studies showing high success. A clot-binding peptide was already used in the surface of micelles to target blood clots, both concentrating an imaging dye and specifically delivering a thrombin inhibitor (Peters et al., 2009). Other authors have used a formulation with a perfluorocarbon core surrounded by a lipid coat, which was derivatized with PPACK (phenylalanine-proline-arginine-chloromethylketone), delivering that thrombin inhibitor to the kidney (Chen et al., 2015). Due to the chemical properties of the core of the NP, it was possible to run quantitative molecular imaging *in vivo* with fluorine MRI, confirming the concentration of particles in the kidney, thrombin binding, and perfusion recovery. The same combined approach was used twice, in the first demonstrating that paramagnetic perfluorocarbon nanoparticles could be used for the non-invasive detection and delineation of a marker of aortic plaque angiogenesis, as well as the local delivery of an effective single treatment of fumagillin, inhibiting plaque angiogenesis at a dose several orders of magnitude lower than previously reported (Winter et al., 2006, 2008). This type of approach becomes especially relevant when the disease severity is rapidly advancing, as the targeted local administration of antiangiogenic agents delays plaque progression and enlarges the window of opportunity for clinical intervention through other conventional methods. The same authors demonstrated that α_V_β_3_-targeted fumagillin NPs could also work synergistically with other therapeutic agents, greatly increasing a continuous clinically relevant antiangiogenic effect (Winter et al., 2008).

This class of combined-effect NPs are now tailoring the future of new therapeutics, with most significance in the administration of therapeutic agents as the disease is being diagnosed, providing a first line of care to the patient. In fact, both the NPs mentioned in the imaging and in the treatment sections may be further manipulated to also acquire the other applicability.

## Conclusion

Nanomedicine is considered as, at least, one of the most relevant paths for the future of therapeutics. This perception has dramatically increased with the new paradigm of personalized medicine. Inserted in this category, NPs with activity toward the diseases responsible for the major death tolls worldwide have deserved special attention. A myriad of systems has been proposed in recent years, some of them described above. However, just a small number has reached clinical trials. More studies are necessary to assess the real potential of these nanosystems, and even different formulations need to be considered if we want to tackle cardiovascular diseases, cancer, or multi-resistant infections.

**Figure 5:**
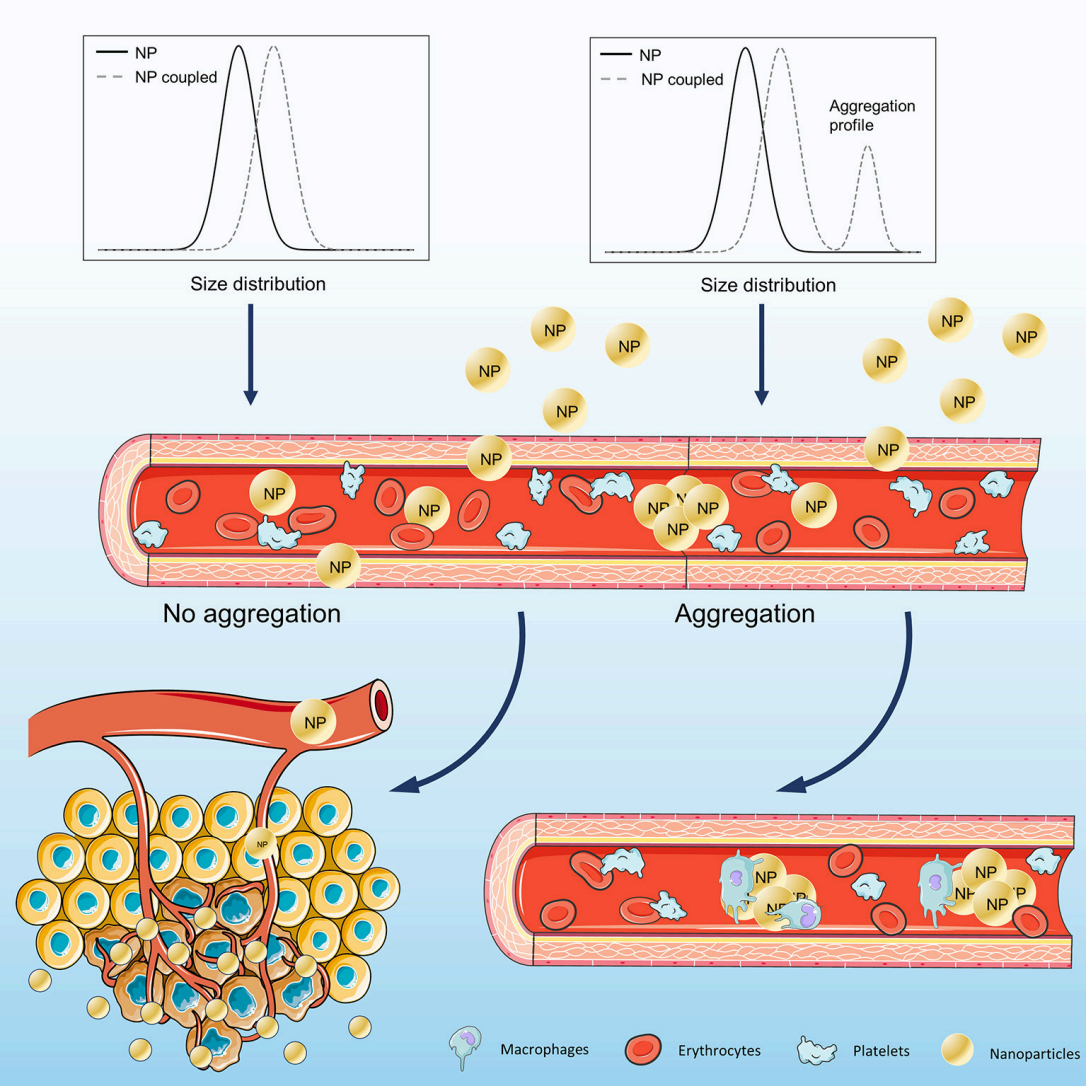
Schematic representation of nanoparticle application in cancer therapy, considering the different size distribution profiles obtained after surface derivatization. NPs may have their surface derivatized with different materials,considering their intended purpose. If the coupled NPs do not aggregate, the size distribution will only reflect an increase corresponding to the coupling material, passing through the blood circulation and acting at the desired targets. Surface derivatization that promotes NP aggregation will be identified in the size distribution. On the bloodstream, NP aggregates will be recognized by macrophages, which will be responsible for their elimination.

Common to all systems described are the methods necessary to characterize the proposed nanoparticles. In this field, light scattering spectroscopy techniques have a considerable number of roles to play, for different purposes. Regarding DLS, it is mostly used to determine the size distribution of the NPs, but some authors use this technique in different ways (Water et al., 2015; Xie et al., 2017; Xia et al., 2018). Confirm surface functionalization, characterize long term stability in different media or pH values, and identification of the aggregation profile are just examples of other possible applications of this technique (Figure 5) (Mohanty et al., 2013; Casciaro et al., 2017; Shmarakov et al., 2017; Zhang et al., 2018). As for zeta-potential, optimization of peptide anchoring profile to the nanoparticle, confirmation of surface charge modification, and validation of electrostatic interaction between the NP and the target cells are some of the processes where it could be essential (Kuai et al., 2010; Takara et al., 2010; Pal et al., 2016). In future studies, light scattering should be essential for the characterization and development of nanoparticles applied to therapeutics, which do not invalidate the fact that other techniques should be also used to further confirm the conclusions obtained.

## Author contributions

All authors listed have made a substantial, direct and intellectual contribution to the work, and approved it for publication.

### Conflict of interest statement

The authors declare that the research was conducted in the absence of any commercial or financial relationships that could be construed as a potential conflict of interest.
